# Glutamine versus Ammonia Utilization in the NAD Synthetase Family

**DOI:** 10.1371/journal.pone.0039115

**Published:** 2012-06-15

**Authors:** Jessica De Ingeniis, Marat D. Kazanov, Konstantin Shatalin, Mikhail S. Gelfand, Andrei L. Osterman, Leonardo Sorci

**Affiliations:** 1 Sanford-Burnham Medical Research Institute, La Jolla, California, United States of America; 2 A. A. Kharkevich Institute for Information Transmission Problems, Russian Academy of Sciences, Moscow, Russia; 3 Department of Biochemistry, New York University School of Medicine, New York, United States of America; 4 Faculty of Bioengineering and Bioinformatics, M.V. Lomonosov Moscow State University, Moscow, Russia; 5 Department of Clinical Sciences, Section of Biochemistry, Polytechnic University of Marche, Ancona, Italy; Stanford University, United States of America

## Abstract

NAD is a ubiquitous and essential metabolic redox cofactor which also functions as a substrate in certain regulatory pathways. The last step of NAD synthesis is the ATP-dependent amidation of deamido-NAD by NAD synthetase (NADS). Members of the NADS family are present in nearly all species across the three kingdoms of Life. In eukaryotic NADS, the core synthetase domain is fused with a nitrilase-like glutaminase domain supplying ammonia for the reaction. This two-domain NADS arrangement enabling the utilization of glutamine as nitrogen donor is also present in various bacterial lineages. However, many other bacterial members of NADS family do not contain a glutaminase domain, and they can utilize only ammonia (but not glutamine) in vitro. A single-domain NADS is also characteristic for nearly all Archaea, and its dependence on ammonia was demonstrated here for the representative enzyme from *Methanocaldococcus jannaschi*. However, a question about the actual *in vivo* nitrogen donor for single-domain members of the NADS family remained open: Is it glutamine hydrolyzed by a committed (but yet unknown) glutaminase subunit, as in most ATP-dependent amidotransferases, or free ammonia as in glutamine synthetase? Here we addressed this dilemma by combining evolutionary analysis of the NADS family with experimental characterization of two representative bacterial systems: a two-subunit NADS from *Thermus thermophilus* and a single-domain NADS from *Salmonella typhimurium* providing evidence that ammonia (and not glutamine) is the physiological substrate of a typical single-domain NADS. The latter represents the most likely ancestral form of NADS. The ability to utilize glutamine appears to have evolved via recruitment of a glutaminase subunit followed by domain fusion in an early branch of Bacteria. Further evolution of the NADS family included lineage-specific loss of one of the two alternative forms and horizontal gene transfer events. Lastly, we identified NADS structural elements associated with glutamine-utilizing capabilities.

## Introduction

Nicotinamide adenine dinucleotide (NAD) serves both, as a ubiquitous cofactor in hundreds of redox reactions and as a substrate in a number of regulatory processes related to cell cycle and longevity, calcium signaling, immune response, DNA repair, etc. [1], [2], [3]. Due to its impact on nearly all aspects of metabolism, NAD is essential for survival and several enzymes involved in its biosynthesis have been recognized as potential drug targets [4], [5]. One of these enzymes is NAD synthetase (NADS), which catalyzes amidation of nicotinic acid adenine dinucleotide (NaAD) in the last step of NAD synthesis. NADS was demonstrated to be essential in a number of bacterial pathogens including *Mycobacterium tuberculosis*, *Bacillus anthracis*, *Staphylococcus aureus*, and *Escherichia coli* [5], [6], and it is currently being pursued as a target for antibiotic development [7], [8]. At the same time, relatively rare alternative variants of NAD biosynthetic pathways that bypass the requirement of NADS were described in some Bacteria [9], [10], [11], [12] and in Eukaryotes [13].

**Figure 1:**
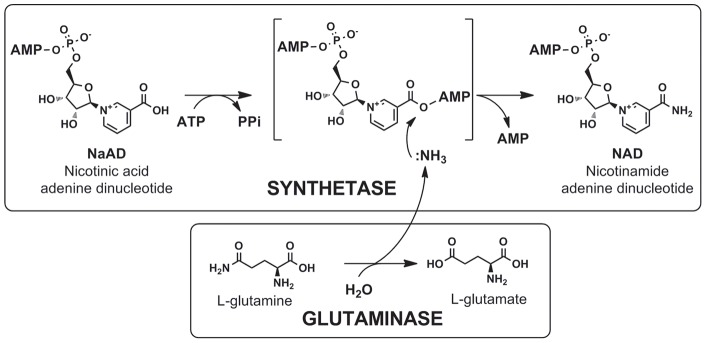
Scheme of the two-step reaction catalyzed by NAD synthetase.

NADS, a member of the N-type ATP pyrophosphatase family [14], catalyzes the ATP-dependent transformation of nicotinic acid adenine dinucleotide (NaAD) into the amide product NAD via a two-step process. In the first step, a pyridine carboxylate group is activated by adenylation followed by amidation via the nucleophilic replacement of the adenylate moiety with ammonia in the second step (Figure 1). This general mechanism involving an adenylation step is shared by other ATP-dependent amidotransferase including GMP synthetase (GuaA), asparagine synthetase B (AsnB) and Glu-tRNAGln amidotransferase (GatABC). Other amidotransferase such as carbamoylphosphate synthetase (CarAB) and formylglycinamidine ribonucleotide amidotransferase (PurL) – both belonging to the ATP-grasp superfamily – and CTP synthetase (PyrG) – belonging to the P-loop NTPase family – also use ATP in their catalytic mechanism, which apparently includes the hydrolysis of ATP to ADP rather than adenylation followed by AMP release [15] (see Table 1). Another mechanistic feature common for enzymes of this class is the *in situ* formation of ammonia through deamidation of glutamine to glutamate by a committed glutaminase domain (or subunit). The molecule of ammonia is directly channeled from the glutaminase domain to the amidation site in the synthetase domain (we will further refer to them as G-domain and S-domain, respectively) without dissociation to the milieu. A compact two-domain arrangement allows these enzymes to utilize glutamine *in vivo* (whereas *in vitro* they can use both, glutamine and ammonia). This ability is of utmost physiological importance, as the cellular level of free ammonia is typically quite low due to its efficient capturing by glutamine synthetase. The latter enzyme was historically considered as the only ATP-dependent amidotransferase that utilizes ammonia (and not glutamine) *in vivo* [16].

**Table 1:** Genomic arrangement of the synthetase and glutaminase components in NAD synthetase and other families of ATP-dependent amidotransferases.

Enzymes	Gene names^1^	Pathway	Glnse class^2^	Arrangement of synthetase and Glnse components^3^				genomes
				Fusion	Cluster	Remote	No Glnse	
NAD synthetase	*nadE*	NAD	IV	54	1	1	44	886
Asparagine synthetase B	*asnB*	Asn	II	100	-	-	-	700
CTP synthetase	*pyrG*	CTP	I	100	-	-	-	800
GMP synthetase	*guaA*	GMP	I	100	-	-	-	730
Formylglycinamidine ribonucleotide amidotrans.	*purL*	Purines	I	52	25	23	-	660
Anthranilate synthetase	*trpDE*	Trp	I	5	70	25	-	570
Carbamoyl-phosphate synthetase	*carAB*	Pyrimidines	I	1	72	27	-	600
Glu-tRNA^Gln^ amidotransferase	*gatABC*	Translation	III	0	67	33	-	490

1 Gene names are as in *E. coli* except *gatABC* and *yaaDE* that are as in *B. subtilis* (not present in *E. coli*). ^2^ A current classification of glutaminase domains (subunits) of amidotransferases includes four classes of nonhomologous enzymes: Class I (contains a catalytic triad in the active site); Class II (contains a catalytic Cys at the N-terminus); Class III (a relatively poorly explored glutaminase subunit of GatABC complex); and Class IV (nitrilase-like glutaminase component of NADS). ^3^ Percentage (%) of total number of arrangements within analyzed genomes.

A similar two-domain arrangement is characteristic of NADS enzymes that are present in Eukaryotes and in many (but not all) Bacteria. The N-terminal G-domain of NADS belongs to the nitrilase family of amidohydrolases [17]. It is structurally distinct from functionally equivalent domains (or subunits) of other ATP-dependent amidotransferases (see Table 1). The role of the G-domain in the NADS mechanism of action was originally described for the yeast enzyme (Qns1) [18], [19] and later confirmed for its human [20] and bacterial orthologs [10], [21], [22], [23]. A series of 3D structures of a glutamine-utilizing NADS from *M. tuberculosis* provided mechanistic insights into functional coupling of its glutaminase and synthetase activities [22], [24].

On the other hand, many bacterial and nearly all archaeal genomes lack a “long” (two-domain) form of NADS and, instead, harbor a “short” NADS (e.g. as encoded by *nadE* gene in *E. coli*), which includes only a core S-domain. Multiple representatives of this single-domain NadE subfamily have been cloned and characterized from various microbial sources both enzymatically [25], [26], [27], [28], [29] and structurally [30], [31], [32], [33], [34], [35]. All these studies, including a first report on an archaeal NADS representative from *Metanocaldococcus jannaschi* in this work, demonstrated that single-domain NADS can efficiently catalyze ATP-dependent conversion of NaAD to NAD *in vitro* using only ammonia but not glutamine.

These observations pose a fundamental question about the *in vivo* source of the amide group for the NADS reaction catalyzed by members of the single-domain NadE subfamily. At least two possibilities are considered: (i) *in vivo* utilization of free ammonia, which was previously considered a unique feature of glutamine synthetase; (ii) *in situ* generation of ammonia from glutamine by a committed (but yet unknown) glutaminase subunit, which is quite common in other ATP-dependent amidotransferase families (Table 1).

In the present study, by comparative genome analysis of ∼ 800 prokaryotic genomes followed by focused experimental verification we demonstrated that the vast majority of single-domain NADS uses free ammonia *in vivo* as amide donor. We also found that a few bacterial species encode a two-subunit form of NADS endowed with glutamine-utilizing activity. Furthermore, in an attempt to generalize our findings and improve NADS classification, we identified sequence motifs discriminating between single-domain (ammonia-utilizing) and two-domain or two-subunit (glutamine-utilizing) NADS subfamilies. Finally, we propose an evolutionary scenario where a single-component form (ammonia-utilizing) of NADS represents the most likely ancestral form of NADS.

## Materials and Methods

### Materials and growth conditions

All bacterial strains and plasmids used in this study are listed in Table S1. Selected mutant strains of *Salmonella typhimurium* LT2 deficient in ammonia utilization (as described in [36]) were kindly provided by Dr. Sidney Kustu (UC Berkeley). Strains were grown in Luria Bertani (LB) or minimal media (9) supplemented with glutamine (5 or 20 mM) or ammonia (20 mM). For DNA manipulations, restriction endonucleases were purchased from Fermentas. DNA was amplified using Pfu Ultra II Fusion DNA polymerase (Stratagene). Plasmids were isolated using the Wizard Plus SV Miniprep Kit (Promega), and PCR products were purified using the Wizard SV Gel and PCR Clean-Up System (Promega). Primers used for molecular cloning and sequencing (including those for the sequencing of the *nadE* gene from nit mutants of *S. typhimurium)* are listed in Table S2. Sequencing of gel-purified DNA amplification products was performed by Eton Bioscience.

### Cloning, heterologous expression and protein purification

The *nadE* gene encoding NADS of *S. typhimurium* LT2 (locus tag: STM1310, *st*_NADS), wild-type and *nit* mutant strains were amplified by polymerase chain reaction from genomic DNA and cloned in pET15b vector as a fusion with an N-terminal His_6_-tag (Novagen). A two-gene operon from *Thermus thermophilus* HB27 encoding a predicted two-component *tt*_NADS comprised of the core synthetase or S-subunit (TTC1538) and a putative glutaminase or G-subunit (TTC1539), was cloned in a pET-derived vector [37] adding His_6_-tag to the N-terminus of the first gene of the operon. Individual genes encoding S-subunit of *tt*_NADS and *mj*_NADS from *Metanocaldococcus jannaschi* DSM (MJ1352) were cloned in the same vector. All recombinant proteins were overexpressed in *E. coli* BL21/DE3 and purified to homogeneity using standard protocols, e.g. as described in [38]. Typically, cells were grown in LB medium to OD_600_ of ∼0.8–1.0 at 37°C, induced by 0.2 mM IPTG, and harvested after 12 h of shaking at 20°C. Proteins were purified from 1-6 L cultures by chromatography on a Ni-NTA agarose column followed by gel filtration on a HiLoad Superdex 200 16/60 column (Pharmacia) with an AKTA FPLC system.

### Activity assays and steady-state kinetic analysis

A continuous assay for NADS activity was based on enzymatic coupling of NAD production (from NaAD and ammonia or L-glutamine) with its conversion to NADH by alcohol dehydrogenase and monitored at 340 nm (*ε*=6.22 mM^−1^ cm^−1^) as previously described [21]. The specific activity assays were carried out at 37°C in a buffer containing 10 mM MgCl_2_, 46 mM ethanol, 16 mM semicarbazide (or 2 mM NaHSO_3_ in case of glutamine utilization), 100 mM HEPES, pH 7.5, in the presence of saturating concentrations of all substrates, 2 mM ATP, 2 mM NaAD, and 4 mM NH_3_ (or Gln). A direct HPLC-based assay was used to assess substrate specificity of thermostable *mj*_NadE. The reaction mixture contained 100 mM HEPES, pH 7.5, 10 mM MgCl_2_, 2 mM ATP, 2 mM NaAD (or NaMN), and 4 mM NH_3_ (or Gln). After 30 min of incubation at 70°C, protein was removed by micro-ultrafiltration using Microcon YM-10 centrifugal filters (Amicon), and filtrates were analyzed on 50×4.6 mm C18 column (Supelco) as described [11].

To determine the steady-state rate constants, two substrates (e.g. NaAD and ATP) were kept constant at saturating concentration, whereas the third substrate (e.g. ammonia or glutamine) was varied over a relevant concentration range. For example, ammonia ranged from 0.1 to 4 mM for wild type *st*_NadE, and from 1 to 40 mM for the mutant *st*_NadE. Measurements were performed in triplicates, and initial velocity data were fitted to the standard Michaelis-Menten equation using GraphPad Prism software package to obtain *K*_m_ and *k*_cat_ values.

### Quantitative RT-PCR analysis

Total mRNA was isolated from *S. typhimurium* LT2, nit11 and SK51 cells grown in either rich (LB) or minimal media plus 5–20 mM glutamine or 20 mM ammonia using the SV total RNA isolation system (Promega). Reverse transcription was performed in the presence of random primers, after DNase treatment (Promega), using SuperScript II reverse transcriptase (Invitrogen). In order to check for DNA contamination, control reactions were carried out in the absence of reverse transcriptase. Real-time PCR was performed using SYBER GreenER qPCR supermix universal (Invitrogen) and specific primers listed in Table S2 amplifying short region of NAD synthetase gene (*nadE*) and *gapA* that was used as internal standard. Amplification conditions were as follows: 15 min at 95°C; 30 s at 95°C, 1 min at 56°C, 30 s at 72°C for 40 cycles and were carried out in a Stratagene Mx3000.

### Bioinformatics analyses

Phylogenomic distribution and genomic context of NADS-encoding genes (fusion events, chromosomal clustering, and co-occurrence of glutaminase and synthetase components) were analyzed using a collection of over 800 completely sequenced and annotated genomes in the SEED database [39] (see Table 1 and Table S3). For comparative purposes, a similar (albeit less detailed) analysis was performed for other families of ATP-dependent amidotransferases and these data are also included in Table 1. A species tree constructed based on the 16S rRNA marker was taken from the MicrobesOnline resource [40]. Annotated NADS genes were loaded into an in-house Oracle XE database [41] and mapped into the species tree using ad-hoc PL/SQL scripts. Multiple sequence alignments of S- and G-domains were constructed using Muscle [42] by the following procedure: (i) first, all sequences were clustered into compact groups based on an approximate tree constructed from a multiple alignment obtained by a conventional procedure. Phylogenetic trees were built by FastTree [43] and the topology of the trees was additionally confirmed using RAxML [44]; (ii) poorly aligned blocks, the regions with numerous gaps, were cut out from the multiple alignments of each gene cluster; (iii) all clusters were merged into one alignment by the profile-to-profile aligning method incorporated in Muscle. Species and gene trees were visualized and color-highlighted using Dendroscope [45]. For the purpose of evolutionary signature analysis the second step in this procedure was omitted. An ancestral character reconstruction was performed by the maximum parsimony method from the Mesquite package [46]. Structural Modeling of the *T. thermophilus* 3D structure was obtained by Modeller [47] using the Chimera interface [48]. Contacts between synthetase and glutaminase domains were recognized using Chimera with default parameters (van der Waals surface overlap ≥ −0.4 Å).

## Results

### NADS family phylogenomic distribution and classification

Among ∼ 940 complete genomes included in the manually curated metabolic subsystem “NAD and NADP metabolism” [49] in the SEED database [39], at least one form of NADS was found to be present in 886 genomes (94%) including 814 bacterial, 56 archaeal and 16 eukaryotics genomes (Table S3). Among 56 species lacking NADS are those with rare NADS-independent variants of the NAD synthesis, such as *Haemophilus influenzae* [50] and *Francisella tularensis* [11], and some obligate intracellular endosymbionts, such as *Chlamydia* and *Rickettsia*, where the entire NAD biosynthetic machinery is replaced by a unique ability to salvage NAD from the eukaryotic host cell [49].

**Figure 2:**
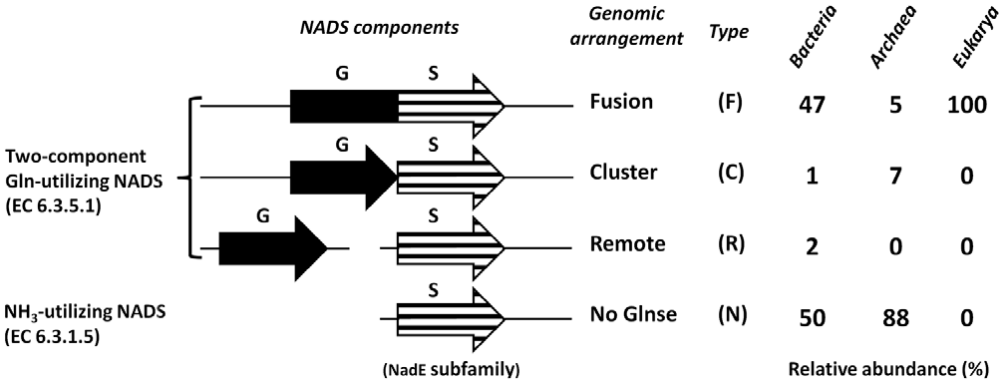
Genomic arrangements of functionally coupled glutaminase (GAT) and synthetase (NADS) components. Comparative genome analysis revealed 4 different genomic arrangements of GAT and NADS components: a) two domain organization (fusion); b) physical clustering; c) remote occurrence; d) absence of glutaminase. Their relative distribution across Archaea, Bacteria, and Eukaryotes is also shown (left side).

All NADS-encoding genes can be classified as one of the two forms: the two-domain form comprised of the N-terminal synthetase (S-domain) and C-terminal glutaminase (G-domain), and the single S-domain form lacking the G-domain (Figure 2). The first form is characteristic of all Eukaryotes and many diverse bacterial (but not archaeal) lineages (termed here *type F* for Fused). The second form was further classified to three types depending on the presence or absence and chromosomal arrangement of a distinct gene encoding a putative G-subunit. The latter was identified by close homology with the G-domain of *type F* NADS in a limited number of bacterial and archaeal species, either clustered on the chromosome with a single-domain S-subunit (termed *type C* for Clustered) or located remotely (*type R* for Remote). However, in the overwhelming majority of genomes encoding a single-domain NADS (such as NadE in *E. coli*) no candidate G-subunit could be detected (termed *type N* for None) (Figure 2 and Table S3).

As already mentioned, all previously characterized *type F* NADS enzymes were shown to efficiently utilize glutamine (in addition to ammonia) for the NaAD transamidation *in vitro*. Based on an analogy with several other ATP-dependent amidotransferase families (Table 1) that can accommodate all three types (F, C and R) of the genomic arrangement for their glutaminase components (domains or subunits), we hypothesized that at least *type C* (and possibly *type R*) NADS enzymes can form a glutamine-utilizing complex comprised of S- and G- subunits. To test this hypothesis we cloned, expressed, purified and performed enzymatic characterization of the predicted two-component (*type C*) NADS from *Thermus thermophilus* HB27 (*tt*_NADS) comprised of S- and G-subunits encoded within a single operon (see below).

The glutamine-utilizing ability of *type F, C* (and likely *R*) NADS is consistent with the anticipated role of glutamine as a universal *in vivo* source of the amide group for most ATP-dependent amidotransferases including NADS (Table 1). At the same, this question remained open for *type N* enzymes. Multiple representatives of the *type N* NADS from diverse bacterial species were previously characterized as strictly ammonia-utilizing *in vitro*. *Type N* NADS enzymes characteristic of Archaea could be tentatively assigned as ammonia-dependent, but to our knowledge, this conjecture has not been experimentally tested prior to this study. To fill-in this knowledge gap we have cloned, expressed, purified, characterized and confirmed ammonia-dependence of NADS from *Methanocaldococcus jannaschii* (see below).

Our exhaustive genome context analysis [51] (including the analysis of operons, regulons, co-occurrence profiles combined with distant homology-based functional assessment) failed to identify any novel (nonorthologous) types of the G-subunit in neither Bacteria nor Archaea. This observation supports (albeit does not prove) a hypothesis originally derived from the genetic study in Salmonella that NadE is directly utilizing free ammonia as a nitrogen donor [16]. This study showed that a growth phenotype caused by mutations in the *nadE* (*nit*) locus could be compensated by increasing ammonia (but not glutamine) level in the media. To test whether the observed phenotype is indeed due to the impaired ability of NadE to directly utilize ammonia rather than pair up with an unknown glutaminase, we aimed to clone, express, purify and characterize the NadE enzyme from the *Salmonella nit* mutant (see below).

The capability to directly utilize ammonia *in vivo* for the amidotransferase reaction would make the NadE subfamily only a second known case after glutamine synthetase. To assess whether this feature could be generalized beyond a single species, we performed a more detailed comparative phylogenetic and evolutionary analysis of the NADS family (as described below).

### A two-subunit NADS from *T. thermophilus* (*tt*_NADS) can utilize glutamine as a nitrogen donor

**Figure 3:**
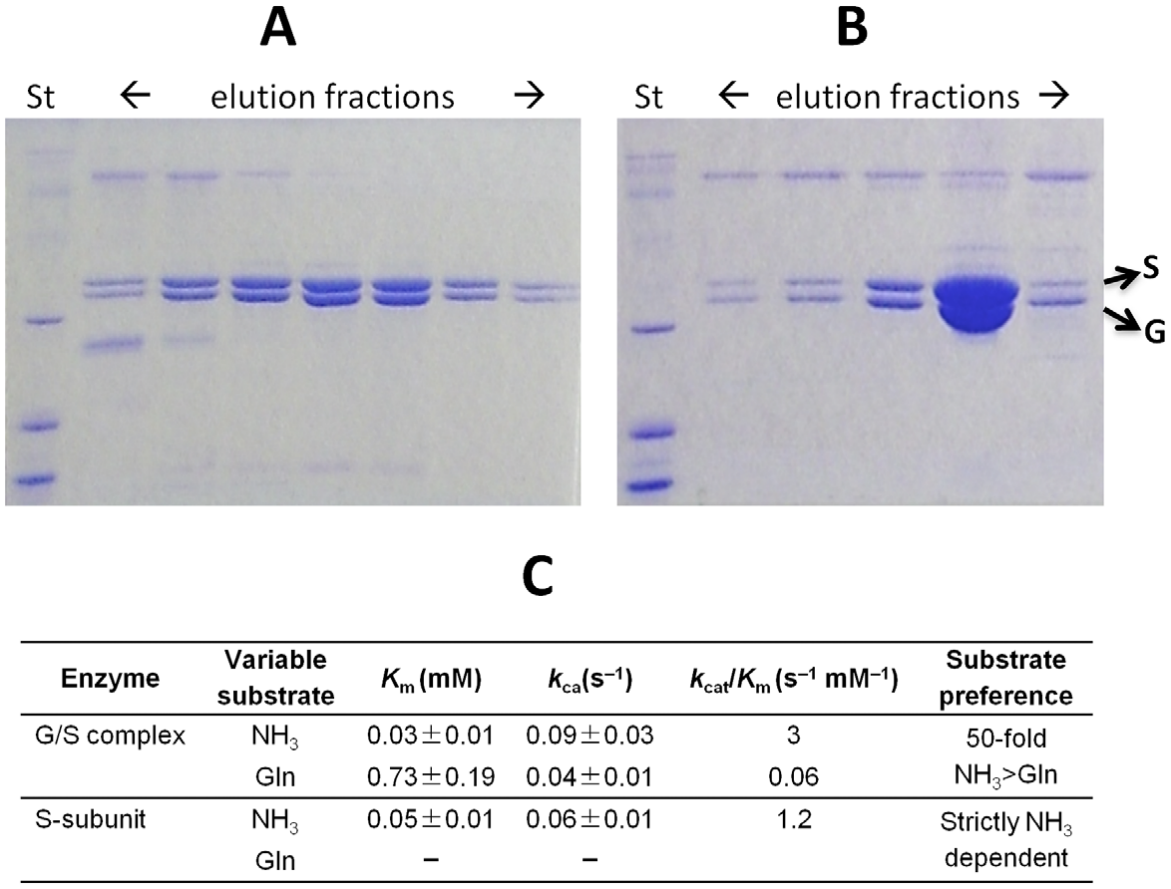
Biochemical characterization of *T. thermophilus* glutaminase and NAD synthetase subunits. SDS-page analysis of Ni-NTA affinity column (A) and gel filtration chromatography (B) elution fractions show that His-tagged recombinant *T. thermophilus* NADS and untagged GAT tend to co-purify. (C) Kinetic characterization of *T. thermophilus* S-subunit and G/S complex.

The co-expression in *E. coli* of the two genes forming an operon (TTC1538 – TTC1539) in *T. thermophilus* showed that their products encoding putative S- and G- subunit of NADS form a tight complex and tend to co-purify on Ni-NTA (Figure 3A) and gel filtration (Figure 3B) chromatography. The SDS-PAGE analysis is suggestive of 1∶1 stoichiometry. The enzymatic characterization of this complex confirmed its NADS activity and the ability to use both, ammonia and glutamine as amide donors. In contrast, the S-subunit alone, when expressed and purified as a single gene, had a comparable NADS activity only with ammonia (Figure 3C) showing *K*_m_ values very close to typical bacterial NadE enzymes [25], [52]. Notably, even for the two-subunit (G/S) enzyme, the substrate preference was still in favor of ammonia over glutamine (∼50-fold), suggesting a theoretical possibility of using both substrates *in vivo*. On the other hand, the preference for ammonia over glutamine reported for a typical NadE enzyme (e.g. ∼ 2,500 fold for the NadE from *Pseudomonas sp.* [25] is substantially higher. In fact, even a low NadE activity observed with glutamine is likely due to its spontaneous hydrolysis and/or some contamination by free ammonia. Overall, the obtained results provided the first experimental verification of the predicted *type C* (and, possibly, *type R*) glutamine-utilizing NADS enzymes (Figure 2).

### A single-domain NADS from *M. jannaschii* (*mj*_NADS) uses ammonia as a nitrogen donor

Members of the NadE subfamily are found in Archaea but no study on a NADS enzyme from an archaeal representative has been reported so far. In fact, the very existence of NAD synthetase function in Archaea could be questioned as in all of them (50 out of 50 genomes analyzed) a typical bacterial NadD enzyme (converting the NaMN precursor to the NaAD substrate of NADS) is replaced by a distantly homologous NadM. Members of NadM family (that are also present in some Bacteria), unlike NadD, can effectively utilize an amidated pyridine nucleotide, NMN, thus providing an alternative (NADS-independent) route to NAD. For example, in the bacterial pathogen Francisella tularensis, a replacement of NadD with NadM is accompanied by a functional adaptation of the NadE homolog as NMN synthetase (NMNS), with only a marginal NADS activity [11]. On the other hand, we have reported that archaeal NadM [12] is a bifunctional NMN/NaMN adenylyltransferase, which, at least theoretically, may operate in conjuction with either NMNS or NADS. For example, a bifunctional NadM acts in concert with a conventional (type F) NADS in another unusual bacterial NAD network of Acinetobacter sp. [10]. To clarify the functional activity of the archaeal NadE sub-family, we have cloned and overexpressed in E. coli a gene MJ1352 encoding a putative NADS from M. jannaschii. The kinetic parameters and substrate specificity of the purified recombinant mj_NADS were evaluated with respect to NaAD and NaMN as substrates with ammonia as amide donor (4 mM) and saturating ATP (2 mM). This analysis revealed a robust ammonia-dependent NADS activity (Km for NaAD 0.13±0.02 mM and kcat of 0.20±0.01 s−1). No appreciable NMNS activity and no NADS activity with glutamine as the amide donor could be detected. Overall, this analysis confirmed classification of the archaeal NadE, together with bacterial members of this subfamily, as ammonia-utilizing (type N) NADS.

### Kinetic properties of a mutant single-domain NADS from *S. typhimurium* (*st*_NADS) are consistent with its physiological role in direct utilization of ammonia *in vivo*

**Figure 4:**
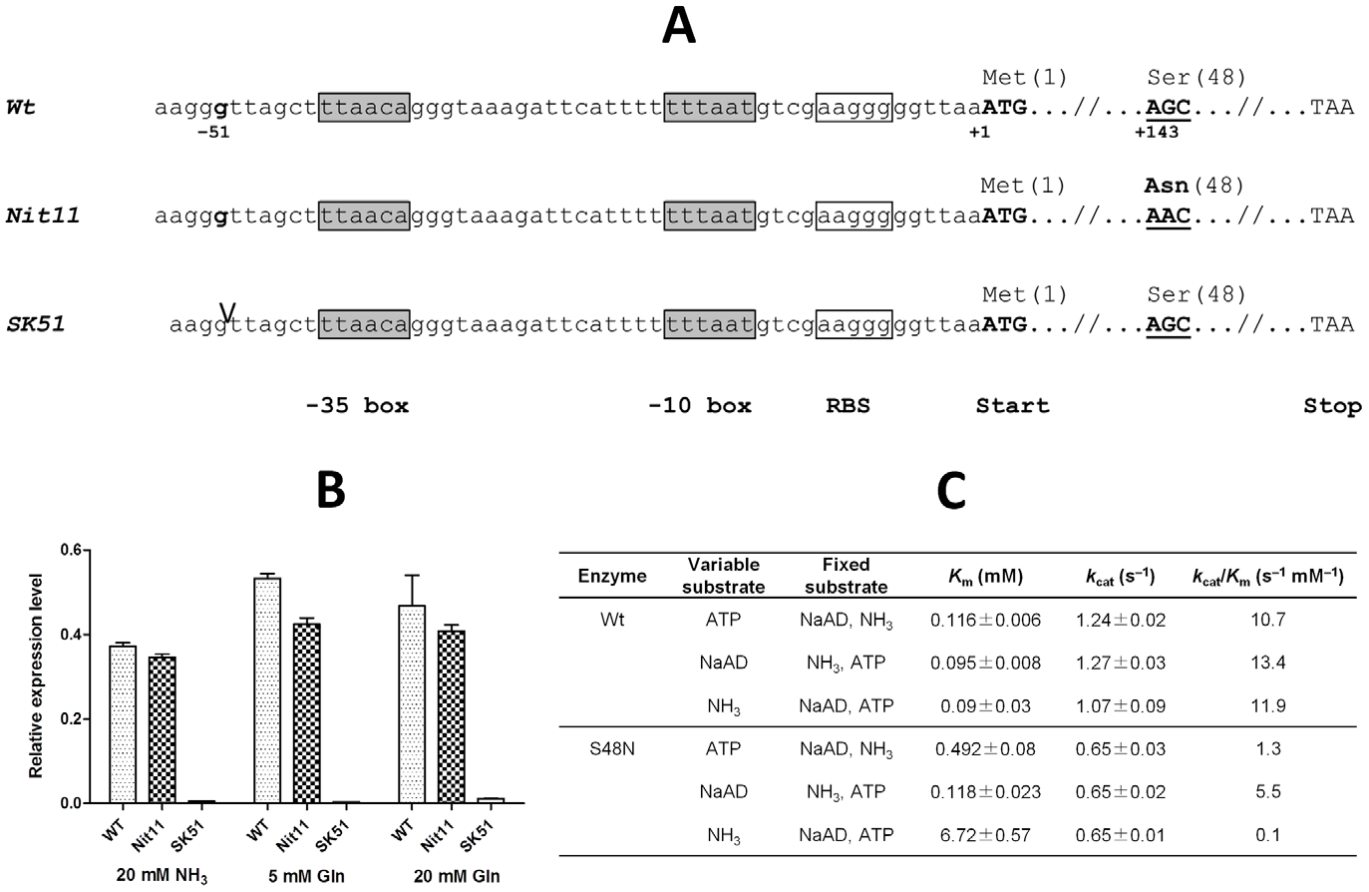
Analysis of Nit11 and SK51 *S. typhimurium* mutant strains. (A) Schematic of *S. typhimurium nadE* mutant strains. Nit11 mutant features a missense mutation at nucleotide 143 yielding the amino acid substitution AN. SK51 mutant features a single nucleotide deletion (G at position – 51 considering as +1 the first base of the start codon) that is just upstream of the -35 box regulatory region of the promoter. Predicted regulatory sequences are indicated in the bottom line. (B) Relative expression level of *nadE* in the wild type and in the two classes of mutants *nit11* (S48N) and SK51 in four different growth conditions: rich medium (1), minimal medium supplemented with 20 mM NH_3_ (2), MM supplemented with 5 mM (3) or 20 mM (4) glutamine. (C) Kinetic characterization of wild type and S48N *S. typhimurium* NAD synthetase. Initial rates were measured by spectrophotometrical coupled (SPEC) assays. The kinetic parameters *K*_m_ and *k*_cat_ are apparent values determined at fixed (saturating) concentrations of co-substrates. For fixed substrates, concentrations were: 2 mM ATP, 2 mM NaAD, and 40 mM NH_3_. Errors represent standard deviation.

*Salmonella nit* mutants that were previously characterized as defective in nitrogen assimilation despite normal levels of ammonia assimilatory enzymes [36], provided us with a valuable case study to further address the question of the *in vivo* amide donor characteristic of NadE subfamily. Whereas both classes of mutants described in the original study, those induced by ICR (SK51) or by nitrosoguanidine (*nit11*), were genetically mapped within the *nadE* locus, the exact nature of these mutations has not been established. Amplification and sequencing of the respective mutant loci revealed two types of genetic lesions: (i) a deletion of a single nucleotide (G) in the promoter region upstream of the predicted -35 box, and (ii) a point mutation at the nucleotide 143 (G to A) replacing Ser-84 residue with Asn (Figure 4A). Therefore, a reported loss of the NADS activity could be expected to occur at the transcriptional level for the first type, and at the level of enzymatic properties for the second type of mutations. Indeed, a quantitative RT-PCR confirmed a substantial (∼ 100-fold) drop of the *nadE* mRNA level in SK51 compared to *nit11* or wild-type *S. typhimurium* (Figure 4B). Importantly, increasing ammonia or glutamine in the media did not have any effect on the relative level of the *nadE* mRNA. It suggests that the observed suppression of growth phenotype is not due to the regulation on the level of transcription, but rather due to overcoming a competition for nitrogen source between a severely suppressed NADS and a robust glutamine synthetase (GlnA). The latter interpretation is consistent with the reported compensatory effect of the *glnA* mutation [36]. To determine the effect of the mutation *nit11* on the enzyme function, wild type *st_*NADS and S48N mutant were overexpressed in *E. coli*, purified and their apparent steady-state kinetic parameters were determined toward ATP, NaAD, and NH_3_ (Figure 4C). The wild-type enzyme had kinetic parameters very close to those reported for *E. coli* NadE [52]. The most dramatic difference from S48N mutant was observed at the level of the apparent *K*_m_ value for ammonia, which was ∼ 70-fold higher for the mutant *st_*NADS compared to the wild-type enzyme. This finding provides a straightforward interpretation for the observed growth phenotype of *nit11* strain, which could be compensated for by increasing the concentration of ammonia in the media [36]. Notably, the mutated residue is a part of the conserved motif SGGXDST characteristic of the N-type ATP pyrophosphatase family [14]. Based on the NadE structure [34], it is located in the vicinity of the active site, which is consistent with the observed effect on the NADS activity.

Therefore, the established effect of mutation on enzyme affinity toward ammonia taken together with the described ammonia-dependence of the respective mutant strain provided additional support to the hypothesis that the single-domain NADS enzymes of the NadE subfamily utilize ammonia, and not glutamine, as a source of the amide group *in vivo*.

### Identification of NADS structural elements associated with glutamine utilization capabilities

**Figure 5:**
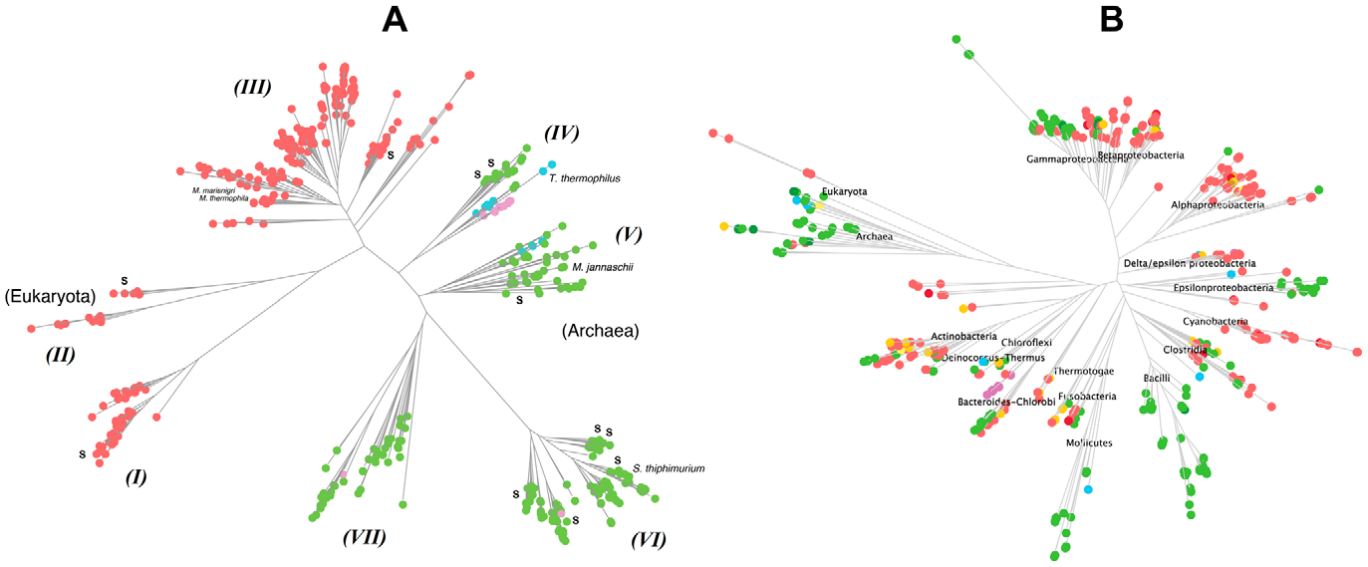
Phylogenetic analysis of NAD synthetase enzyme family. (A) Schematic representation of NAD synthetase phylogenetic tree (full version is in Figure S1) constructed based on synthetase domain. Defined types of NAD synthetase genes – “Fused” (*type F*), “Clustered” (*type C*), “Remote” (*type R*) and “None” (*type N*) are highlighted by red, green, cyan and magenta colors, respectively. The whole tree is partitioned by topology into clusters, which are designated as I–VII branches. (B) Schematic representation of species tree with mapping of NAD synthetase gene types (full version is in Figure S2). Genomes containing single NAD synthetase gene of F, N, C, and R types are depicted by red, green, cyan and magenta colors, respectively. Genomes that possess more than one NAD synthetase gene are divided into “multiple F”, “multiple N”, “single F – single N” and “all others” genome groups, which are highlighted by dark red, dark green, orange and yellow colors, respectively.

A projection of the classification described above onto the NADS family phylogenetic tree (Figure 5A, for details see Figure S1) shows remarkable consistency for the three branches I–III covering all enzymes of the *type F*. While most of NADS sequences within branches IV–VII belong to the *type N*, representatives of the *type C* (and *R*) are found intertwined among them within branches IV and V. Therefore, sequence-based discrimination between genuine single-component (ammonia-utilizing) NADS and two-subunit (glutamine-utilizing) enzymes represents a challenge. It is particularly important since many genomes contain distant representatives of the nitrilase family with yet unassigned functions that could be considered candidate G-subunits for NADS enzymes presently classified as *type N*.

To address this challenge we aimed to identify the hypothesized signature sequence elements (motifs) characteristic of two-component glutamine-utilizing NADS enzymes distinguishing them from single-component ammonia-utilizing enzymes. Identification of such motifs would help to generalize our findings and improve the NADS classification. For this purpose we combined comparative sequence analysis with available 3D structural data. Each of the seven branches of the NADS phylogenetic tree is represented by at least one of 18 reported 3D structures (Figure 5A). Detailed examination of the key residues implicated in substrate, cofactor and metal ion binding sites [22], [30], [31], [32], [33], [34], [35], [53] (Table S4) failed to identify any residues that are distinct between the two groups of NADS enzymes: (i) glutamine-utilizing (*type F, C* and *R*) and (ii) ammonia-utilizing (*type N*), but conserved within each group. Likewise, no residues significantly correlating with the separation of ∼ 800 analyzed sequences between the two groups could be identified by the specificity-determining position prediction method implemented in the SDPpred algorithm [54]. One limitation of this method (which was successfully used by us for functional classification of other protein families, e.g. [55]), is the required elimination of poorly aligned regions containing major gaps. The inspection of such regions removed from NADS multiple alignment showed that they mostly belong to the surface loops that are generally known to accumulate frequent insertions and deletions [56] contributing to the adaptive evolution of protein-protein interactions interface [57].

**Figure 6:**
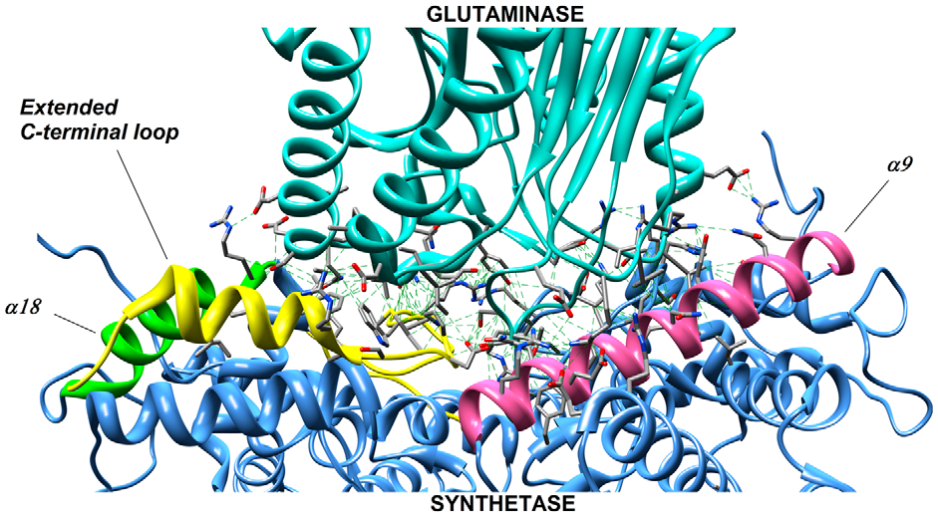
Contact regions between synthetase (blue) and glutaminase (cyan) domains in NADS from *Mycobacterium tuberculosis*. The majority of interacting residues were found in following structural regions-the α9, α18 helices and the extended C-terminal loop, which are highlighted by pink, green and yellow colors, respectively.

We hypothesized that structural elements discriminating between the two NADS groups may reside within the looping regions of enzymes from the first group that are responsible for the interactions between the S- and G-domains (or subunits), and that do not have corresponding regions in the single-component enzymes. Thus, all four structural components of an S-domain comprising an interaction interface with the G-domain: the α9, α18 and α20 helices and an extended C-terminal loop (as identified in the original *M. tuberculosis* NADS structure, Figure 6), appear to play the same role in other reported *type F* NADS structures from *Cytophaga hutchinsonii* (PDB:3ILV), and *Streptomyces avermitiis* (PDB:3N05). The respective regions of the amino acid sequence are conserved in all three branches (I–III) of the phylogenetic tree covering *type F* enzymes. On the other hand, two of these structural elements, the α18 helix and the C-terminal loop, are absent in all five reported 3D structures from the major branch VI representing *type N* single-domain enzymes, including *st*_NADS. A multiple alignment rebuilt to include all variable regions confirms that these structural elements are absent in all representatives of this branch (Table 2 and Figure S2). Of these two elements, the α18 helix is detectable in three reported 3D structures from the branch IV and V. However, none of these structures include a C-terminal loop. The complete multiple alignment suggests that the latter is a signature element distinguishing predicted two-subunit enzymes mapped in these two branches (such as *tt*_NADS in branch IV) from their single-component counterparts (such as *mj*_NADS in the predominantly archaeal branch V, see Figure S3).

**Table 2:** Distribution of two-component glutamine-utilizing NADS signature elements across phylogenetic branches.

branches	α9 helix	α18 helix	Extended C-terminal loop
			
I	+	+	+
II	+	+	+
III	+	+	+
IV	+	+	+/−
V	+	+	+/−
VI	+	−	−
VII	+	−	−

Overall, a mandatory presence of all signature elements listed above (see Table 2) as a necessary and sufficient requirement distinguishing all three types of two-component enzymes from single-component ones was confirmed for nearly all analyzed sequences. The only two exceptions were NADS enzymes from *Vibrio alginoliticus* and *Desulfococcus oleovorans* (branches VI and VII, respectively) originally classified as *type R* based on the presence of a putative G-subunit ortholog in the respective genomes.

### Evolution of the NADS family

A mosaic distribution of different NADS forms over the species tree (Figure 5B, for details see Figure S4) implies a complex evolutionary history of this ancient enzyme family. The most intriguing question is whether a two-component (glutamine-utilizing) or a single-component (ammonia-utilizing) NADS represents the most likely ancestral form. Was it a two-domain form in the Last Universal Common Ancestor (LUCA), which experienced domain fission in the archaeal and some bacterial lineages? Or a single-domain NADS was fused with a nitrilase homolog in one of the deep-branching Bacteria and then passed onto an ancestor of the modern Eukaryotes? The analysis of the NADS phylogenetic tree built from the multiple alignment of S-components only (Figure 5A and Figure S1) shows a complete separation of fused enzymes (*type F*) from all other NADS (*types C, R* and *N*). It suggests that an underlying fusion or fission event could have occurred only once at an early stage of the family evolution. The parallel analysis of the phylogenetic tree built from the multiple alignment of G-components alone (domains and subunits) (Figure S5) revealed a strikingly similar topology with the respective part of the S-component-based NADS tree. This observation points to co-evolution of S- and G-components, and it confirms the conjecture about the unique nature of the ancient fusion/fission event. Further detailed analysis of the NADS tree topology within each kingdom allowed us to reject fission of an ancestral two-domain NADS as an unlikely evolutionary scenario compared to a more likely fusion of ancestral S- and G-subunits. Indeed, a single-domain NADS is a predominant and obviously ancestral form for the Archaea. Few cases of *type F* NADS observed in Archaea (e.g. *Methanocalleus marisnigri, Methanosaeta thermophila*) clearly result from a relatively recent horizontal gene transfer (HGT), most likely from Cyanobacteria. This conclusion was also formally supported by the reconstruction of the ancestral character using the maximum likelihood method (Figure S6). The topology of spreading of NADS forms over diverse groups of Bacteria (Figure 5B) is inconsistent with the origination of either form from a relatively recent HGT. Moreover, an observation that the bacterial single-domain NADS branch IV is the closest to the archaeal branch V (see Figure 5A), suggests that a possible root of the NADS tree and, thus, a hypothetical NADS ancestral form would be single-domain. This conjecture is consistent with the enrichment of branch IV with enzymes from deep-branching thermophilic Bacteria. Notably, most two-subunit (*type C* and *R*) enzymes also belong to the branches IV and V, pointing to an ancestral hypothesized character recruitment of a G-subunit and its operonization with an S-subunit. The subsequent evolution of a two-component form followed by multiple gene loss and acquisition events in different lineages generated the complex taxonomic distribution of NADS forms in Bacteria. A more detailed analysis of this distribution for selected taxonomic groups (those containing *tt*_NADS and *st*_NADS described in this study) is provided in Supporting Information (Text S2 and Figure S7). The topology of the two-domain NADS sub-tree clearly indicates that its eukaryotic branch II originates from the bacterial branch I. This branching point (supported by a bootstrap value of 97%) is sufficiently remote from the possible root arguing in favor of bacterial origin of the ancestral eukaryotic NADS.

**Figure 7:**
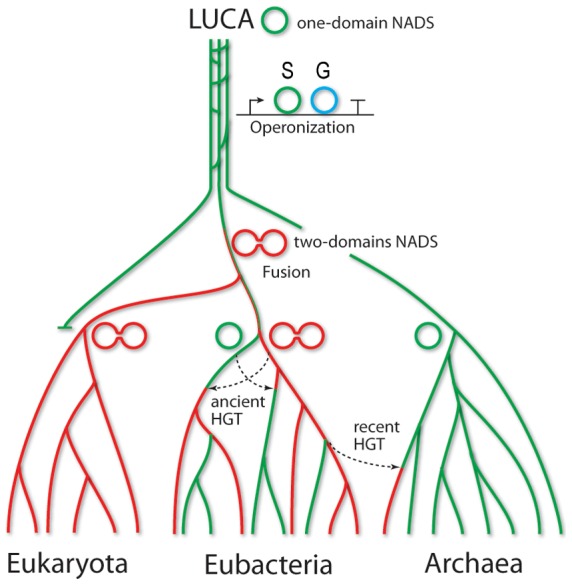
Tentative evolutionary scenario of one- and two-domain form of NAD synthetase enzyme family.

Based on these observations, we propose an evolutionary scenario for the NADS family illustrated in Figure 7. Briefly, an ancestral NADS in the last universal common ancestor (LUCA) was likely a single-domain ammonia-utilizing enzyme. A two-domain glutamine-utilizing form emerged at an early stage of evolution, possibly via intermediate state of clustering of the S- and G-subunits in one operon [58]. It could either happen in Bacteria shortly after separation from Archaea, or before this separation and then lost by the common ancestor of Archaea. The two-domain NADS was acquired by the common ancestor of eukaryotes (LECA, [59]).

## Discussion

In this study we addressed a fundamental question about the physiological donor of the amide group for the ATP-dependent amidation of NaAD to NAD, the last step in the biosynthesis of this ubiquitous redox cofactor, which is catalyzed by divergent members of the NADS enzyme family (Figure 1). In contrast to other classes of ATP-dependent amidotransferases, which are typically comprised of two components (domains or subunits) endowed with synthetase and glutaminase activities, all archaeal and nearly half of the bacterial NADS appear to be comprised of just a single synthetase (S-) domain (Figure 2, Table S3). Multiple representatives of the latter subfamily from a variety of diverse Bacteria (e.g. NadE from *E. coli*) were shown to utilize only ammonia (and not glutamine) as an amide donor in vitro, whereas the situation in the cells remained open for alternative interpretations. Among them, the two most plausible ones were: (i) the existence of a yet uncharacterized glutaminase (G-) subunit (instead of a G-domain) that would allow the NadE-type enzymes to utilize glutamine just as the two-domain NADS and most other ATP-dependent amidotransferases; (ii) the direct utilization of ammonia as the nitrogen donor *in vivo*, which was traditionally considered a unique feature of glutamine synthase (GlnA in *E. coli*). Among the arguments in favor of the first scenario was a presumably limited availability of free ammonia in the cells as well as a conventional two-component arrangement typical for the entire class of ATP-dependent amidotransferases (Table 1). Arguments supporting the second possibility were an apparent absence of candidate genes for G-subunits in the overwhelming majority of the genomes containing single-domain NADS, and indirect experimental evidence obtained by genetic methods in *Salmonella* [16].

In this study we further explored both plausible scenarios by combining bioinformatics with focused experimental analysis. Remarkably, in a comparative genome analysis of over eight hundred prokaryotic genomes, we found several cases of genomic co-occurrence of distinct genes encoding putative orthologs of both, S- and G-domains (Figure 2). The observation that in some cases these genes are located next to each other on the chromosome, hence forming putative operons (*type C* in Figure 2), allowed us to suggest the existence of two-subunit glutamine-utilizing NADS enzymes. This hypothesis was experimentally verified for a complex of S- and G-subunits encoded by the TTC1538-TTC1539 operon from *T. thermophilus.* This finding additionally generalized to a small group of genomes where the putative orthologs of S- and G-subunits occur in remote positions of the genome (*type R*) provides supporting evidence for the first scenario.

However, this scenario cannot be directly applied to nearly half of bacterial genomes that contain no putative orthologs of the G-subunit. Most archaeal genomes also contain orthologs of S-subunit but no orthologs of the G-subunit further expanding the taxonomic distribution of putative single-domain (*type N*) NADS enzymes. Since the experimental characterization of archaeal NADS has not been previously reported, here we filled this gap by confirming ammonia-dependent NADS activity of the representative recombinant protein *mj*_NADS from *M. jannaschii.* Further detailed analysis of the *nadE* genomic context (putative operons) over the entire collection of bacterial and archaeal genomes integrated in the SEED database [39] revealed no plausible candidate gene for an alternative (nonorthologous) G-subunit. This is in contrast with other ATP-dependent amidotransferases whose G- and S-subunits are typically encoded within the same operon (Table 1), arguing in favor of the second possible interpretation, i.e. a direct utilization of ammonia by *type N* bacterial and archaeal NADS enzymes. A similar suggestion was previously reported based on the analysis of *nit* mutants of *S. typhimurium* with deficient nitrogen assimilation (from glutamine and ammonia) due to mutations in the *nadE* locus [16]. Here, we provide further support to this hypothesis by the identification of specific lesions in two selected mutant strains (*nit11* and SK51), and establishing a molecular mechanism underlying the observed phenotype. Indeed, *nit11* featured a mutation of a highly conserved amino acid in the active site of bacterial NAD synthetase (S48N), which impaired the enzyme's ability to use ammonia (*K*_m_ for ammonia increased ∼100-fold compared to the wild type, figure 4C). Based on the published structural analysis, this conserved residue participates in the stabilization of the adenylate intermediate (20), which, according to our data, increased the ammonia requirement in the mutant enzyme. The mutant of the second class, SK51, featured a deletion in the promoter region that dramatically decreased the *nadE* gene expression. Our results suggest that the ability of these mutants to restore normal growth in the presence of high ammonia supplementation is not due to upregulation of gene expression but rather to the increased availability of the ammonia substrate necessary to overcome the competition with a fully active glutamine synthetase (GlnA). Indeed, these *nit* mutations could be suppressed by a mutation in *glnA* gene [36]. Overall, these data strongly support the hypothesis that at least some members of the NadE subfamily directly use ammonia, and not glutamine, as a source of the amide group *in vivo*.

A more detailed comparative sequence and 3D structure analysis of the NADS family allowed us to establish signature motifs that help to distinguish between single-component and less obvious cases of two-component (type R) NADS enzymes. These motifs (α9 and α18 helices, and extended C-terminal loop) comprising the interaction interface between S- and G-domains or subunits are invariantly present in all diagnosed two-component NADS, but at least some of them are absent (or substantially changed) in single-component enzymes (see Table 2 and Text S1). For practical purposes, the identified signature motifs are expected to improve quality of homology-based automated assignment of new members of NADS family as glutamine- or ammonia-utilizing enzymes. This is of particular utility for metagenomic data analysis when the genomic co-occurrence of S- and G-subunits cannot be directly assessed.

The NADS family features a complex phylogenetic distribution pattern of single-component and two-component forms. Thus, Eukarya and Archaea form two compact groups comprised exclusively or almost exclusively of single-domain or two-domain forms, respectively. On the other hand, Bacteria feature a mosaic distribution of both forms pointing to a complex evolutionary history. Based on the combined analysis of the NADS and species trees (Figure 5 and Figure S1 and S2) we propose the following evolutionary scenario (Figure 7): (i) a single-domain ammonia-utilizing NADS was the ancestral form present in the LUCA; (ii) a two-subunit glutamine-utilizing form evolved via recruitment, specialization and operonization of a nitrilase homolog (G-subunit) at an early stage of the NADS-family evolution; (iii) a two-domain form emerged as a fusion of the S- and G-subunits in one of the deep-branching bacterial lineages shortly after separation from Archaea (alternatively it could have emerged before separation but lost by the universal ancestor of Archaea); (iv) Archaea maintained the indigenous single-domain NADS with a few exceptional cases of HGT of a two-domain NADS from Bacteria; (v) the mosaic distribution of single-domain and two-domain forms resulted from a combination of HGT and gene loss without additional fusion or fission events; (vi) Eukarya inherited and adopted the bacterial two-domain NADS. A proposed evolution of the ability to utilize glutamine as an alternative amide donor appears to be reflected in the glutamine vs ammonia preference reported for diverse two-component NADS enzymes. Thus, a presumed ancestral two-subunit form represented by NADS from *T. thermophilus* characterized in this study displays a 50-fold preference for ammonia over glutamine substrate. The NADS from *Thermotoga maritima* representing branch III, the most ancestral of the three branches containing two-domain forms, shows an equal preference for both glutamine and ammonia (40). Interestingly, in addition to the two-domain form, *T. maritima* contains a single-domain NADS of the NadE subfamily supporting a possible physiological relevance of both glutamine and ammonia as NADS substrates. Finally, characterized two-domain enzymes representing the other two, apparently more specialized branches I and II, show a clear preference for glutamine over ammonia, ∼4-fold for NADS from *M. tuberculosis* [22] and ∼6-fold for human [20], [60].

In summary, a comparative genome analysis of NAD synthetase allowed us to predict and experimentally confirm that *i*) ammonia serves as the *in vivo* nitrogen donor for one-domain NADS *ii*) NAD synthetase and glutaminase enzymes, encoded by clustered genes, form a tight functional complex endowed with glutamine-utilizing capability as characteristic of two domain NADS form. These results allowed us to tentatively generalize the existence of a two-subunit glutamine-utilizing NADS toward those bacterial genomes with a remote chromosomal arrangement of synthetase and glutaminase, as further supported by our identification of glutamine-utilization motifs in the extended group of two-components NADS. Lastly, we propose that one-domain ammonia-utilizing NADS was the ancestral NADS form. Bacteria could have engineered the ability to utilize glutamine via an intermediate state of clustering up to the fusion with a recruited nitrilase homolog, an ancestor of the modern G-domain of NADS.

## Supporting Information

Figure S1 **Full NAD synthetase phylogenetic tree constructed based on synthetase domain.** Color scheme is from figure 5A.
(TIF)

Figure S2 **Structural/sequence comparison of the protein segments involved in synthetase-glutaminase interactions.** The alignment is based on NAD synthetase enzymes, and only representatives of the main branches of NADS tree are illustrated. It can be noted the presence of α9 helix in all groups, α18 helix in branches I-V only and the extended C-terminal loop pervasively in branches I–III and eventually in branches IV–V.
(TIF)

Figure S3 **Glutamine-utilizing signature elements in NAD synthetase enzymes from IV–V branches.** The striking correlation of the presence of the extended C-terminal loop with *types C* and *R* enzymes can be observed.
(TIF)

Figure S4 **Full species tree with genome-related mapping of NAD synthetase gene classes.** Color scheme is from figure 5B.
(TIF)

Figure S5 **Phylogenetic tree of the glutaminase domain of NAD synthetase.** Stand-alone GAT genes of C- and R-class NAD synthetase genes were added into analysis. Format of the terminal nodes is the same as for the synthetase domain NADS tree (Figure 5A).
(TIF)

Figure S6 **A reconstruction of ancestral states of NAD synthetase gene forms over species tree.**
(TIF)

Figure S7 **The γ-proteobacteria branch of species tree annotated with suggested HGT and gene loss events.**
(TIF)

Table S1 **Bacterial strains and plasmids used in this study.**
(DOCX)

Table S2 **Primers used for gene sequencing, cloning, and qRT-PCR.**
(DOCX)

Table S3 **Comparative genome analysis of the synthetase and glutaminase components of NADS enzyme family.**
(XLSX)

Table S4 **Key catalytic residues across 18 NADS with available 3D structure.**
(DOCX)

Text S1 **Correlation between predicted glutamine-utilizing property and glutamine-utilizing signature motifs.**
(DOCX)

Text S2 **The mosaic distribution of one and two-domain NAD synthetase in Eubacteria.**
(DOCX)
