# Supplementary figures and images for: Glutamine versus Ammonia Utilization in the NAD Synthetase Family
Source: PLoS One. 2012 Jun 15;7(6):e39115. doi: 10.1371/journal.pone.0039115 (PMC3376133; doi:10.1371/journal.pone.0039115)

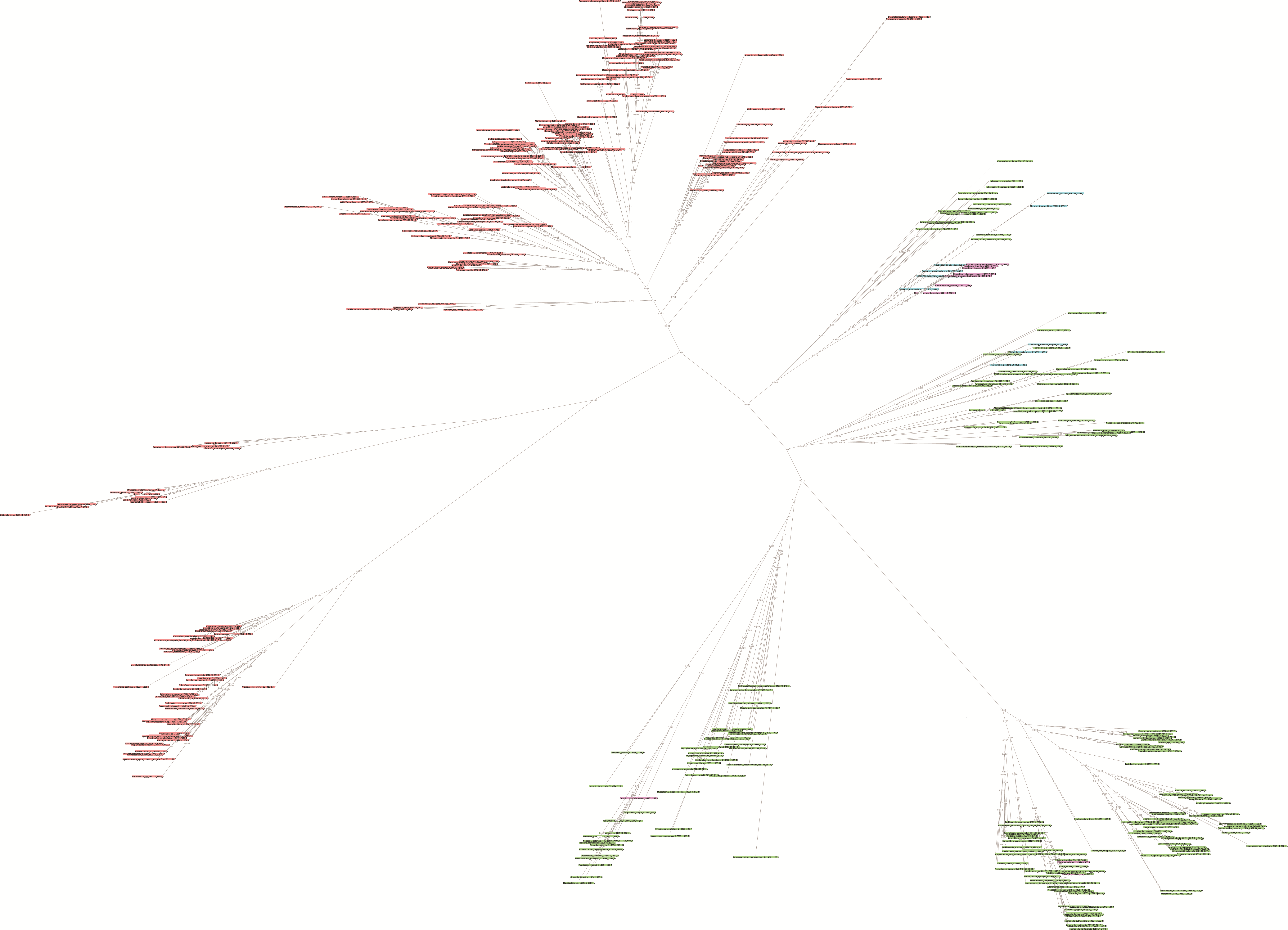

Supplement: Figure S1 — Full NAD synthetase phylogenetic tree constructed based on synthetase domain. Color scheme is from figure 5A. (TIF) [file pone.0039115.s001.tif]

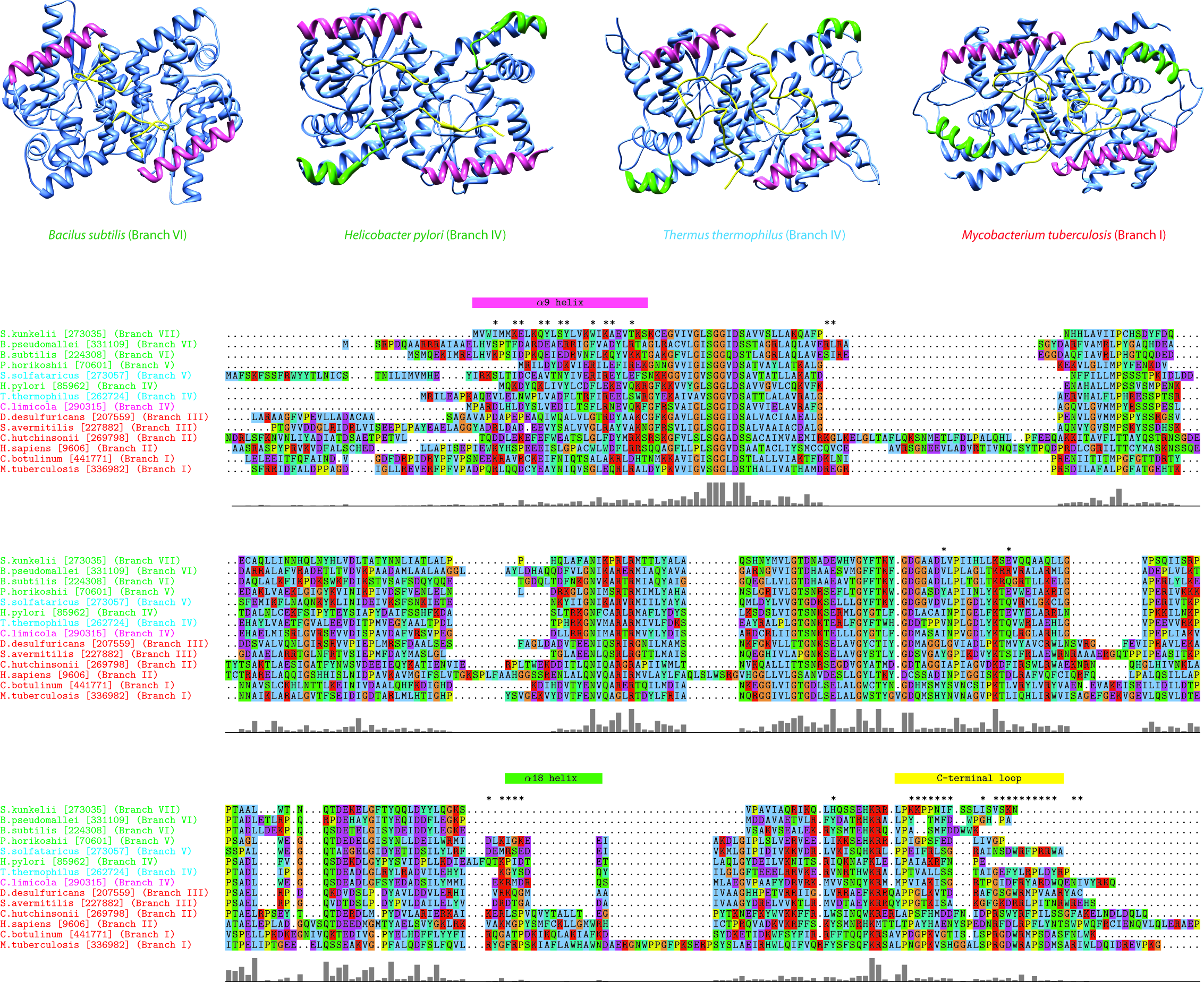

Supplement: Figure S2 — Structural/sequence comparison of the protein segments involved in synthetase-glutaminase interactions. The alignment is based on NAD synthetase enzymes, and only representatives of the main branches of NADS tree are illustrated. It can be noted the presence of α9 helix in all groups, α18 helix in branches I-V only and the extended C-terminal loop pervasively in branches I–III and eventually in branches IV–V. (TIF) [file pone.0039115.s002.tif]

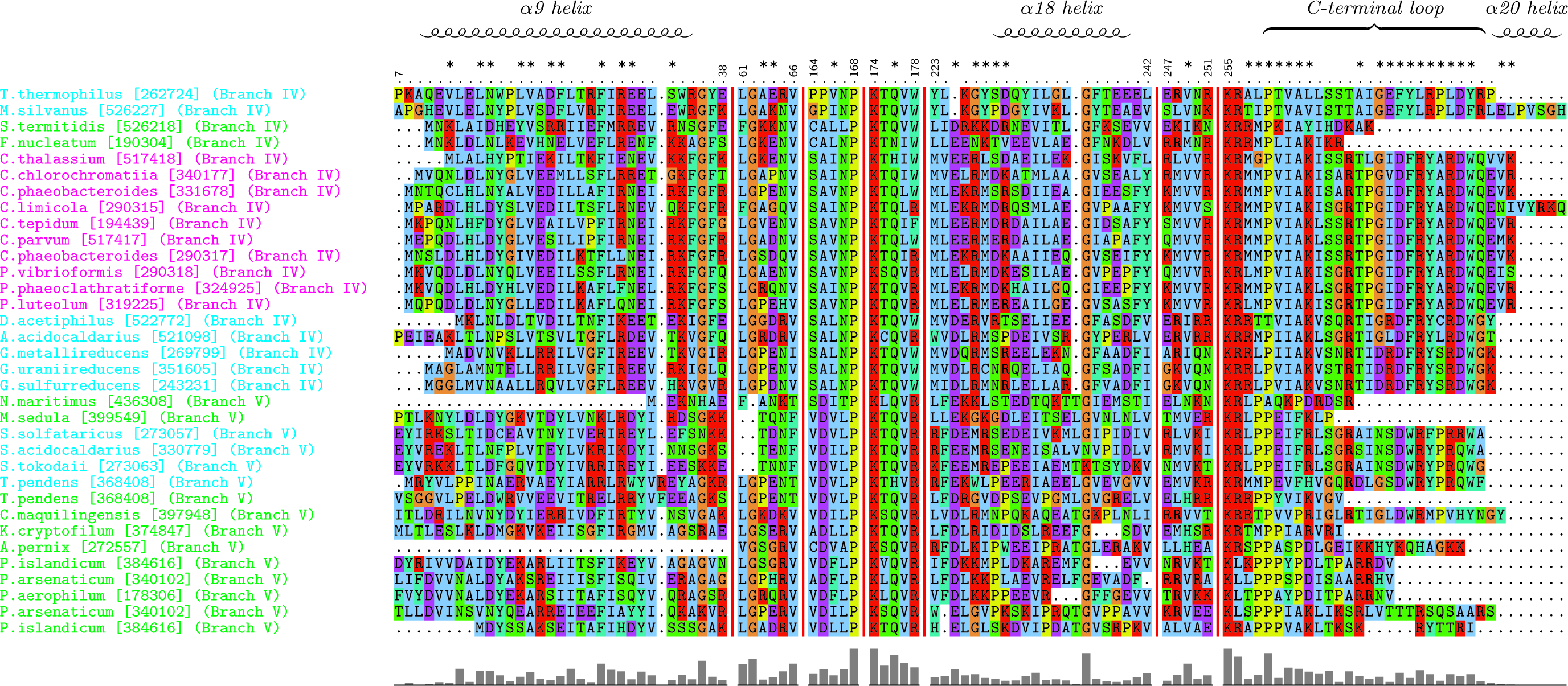

Supplement: Figure S3 — Glutamine-utilizing signature elements in NAD synthetase enzymes from IV–V branches. The striking correlation of the presence of the extended C-terminal loop with types C and R enzymes can be observed. (TIF) [file pone.0039115.s003.tif]

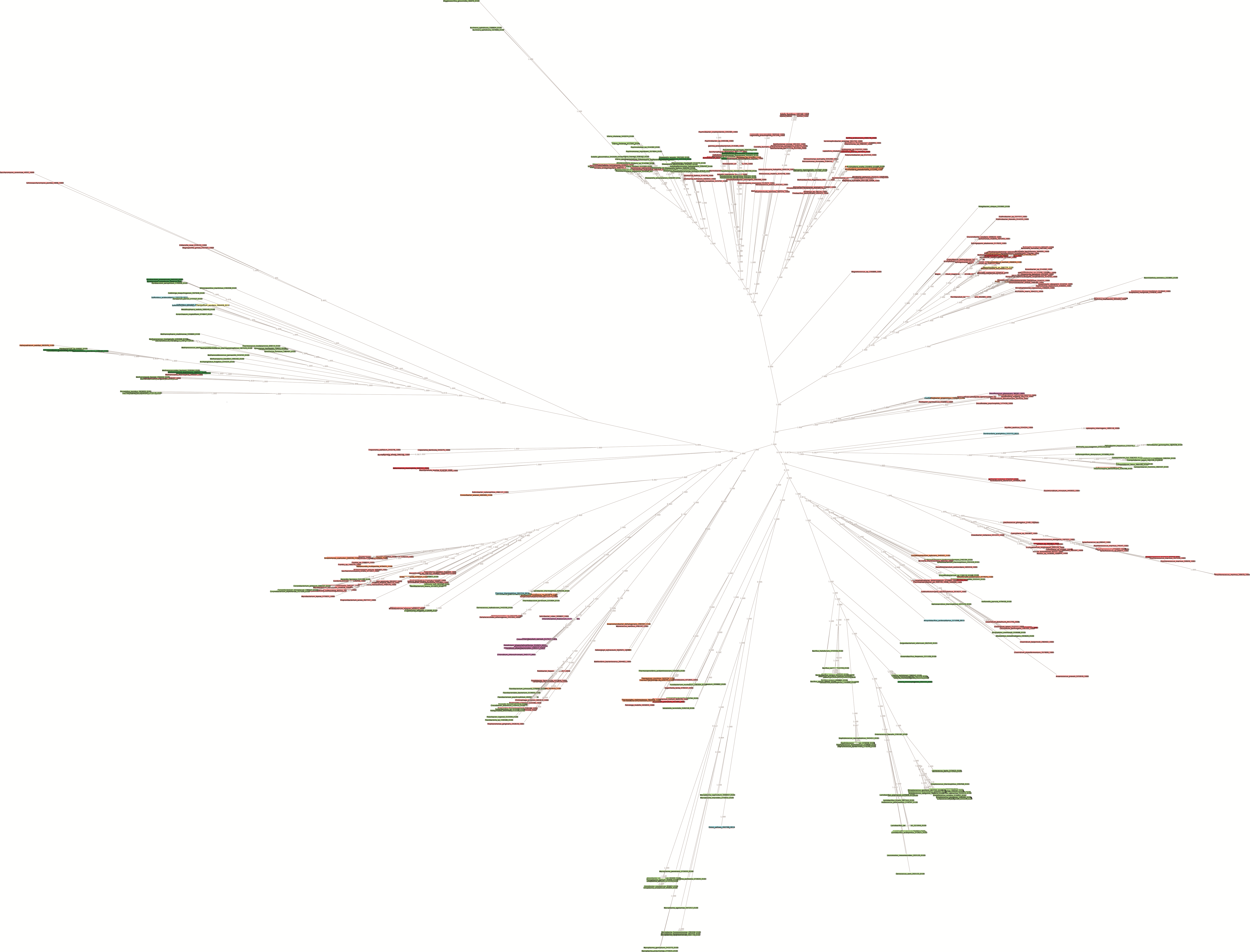

Supplement: Figure S4 — Full species tree with genome-related mapping of NAD synthetase gene classes. Color scheme is from figure 5B. (TIF) [file pone.0039115.s004.tif]

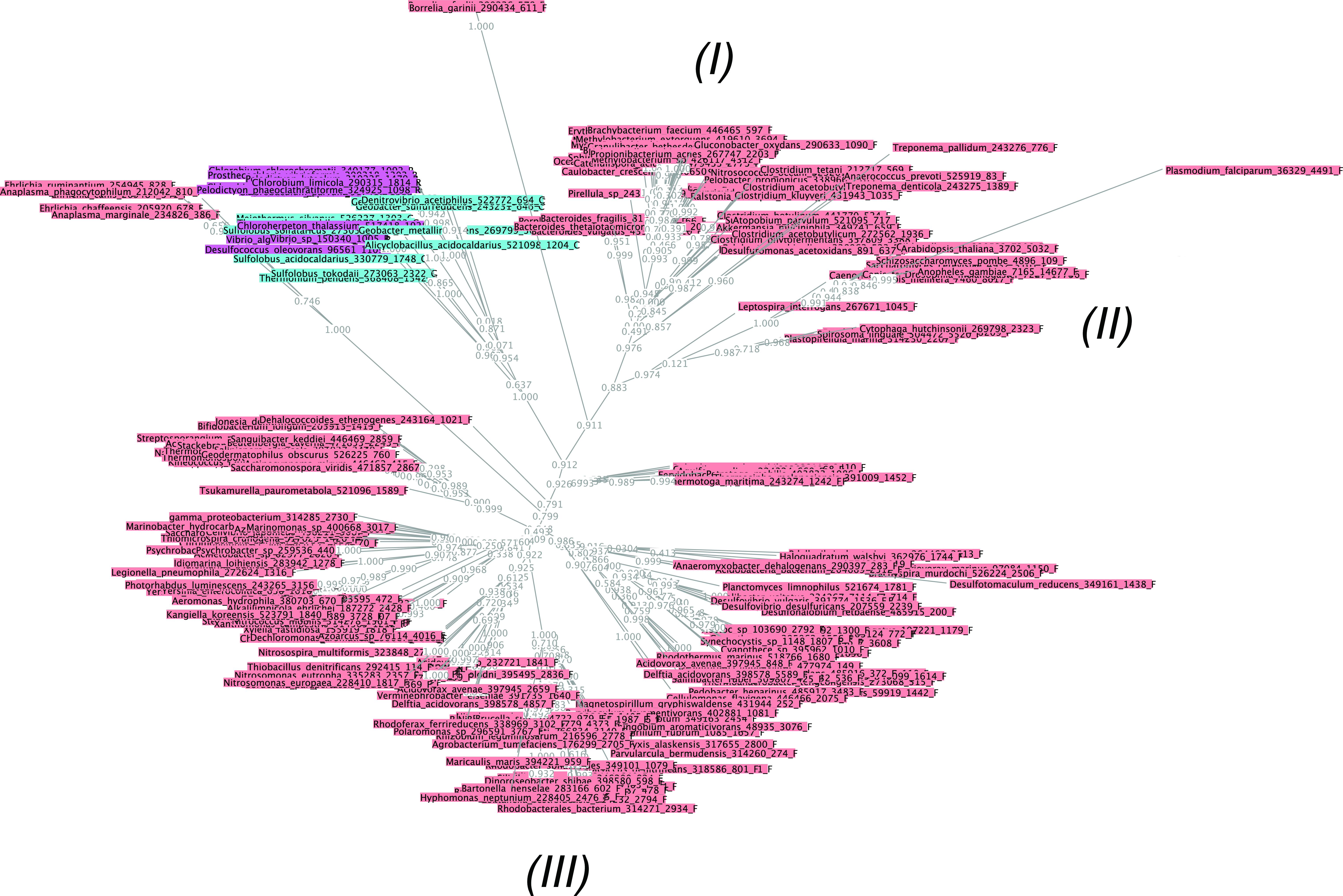

Supplement: Figure S5 — Phylogenetic tree of the glutaminase domain of NAD synthetase. Stand-alone GAT genes of C- and R-class NAD synthetase genes were added into analysis. Format of the terminal nodes is the same as for the synthetase domain NADS tree (Figure 5A). (TIF) [file pone.0039115.s005.tif]

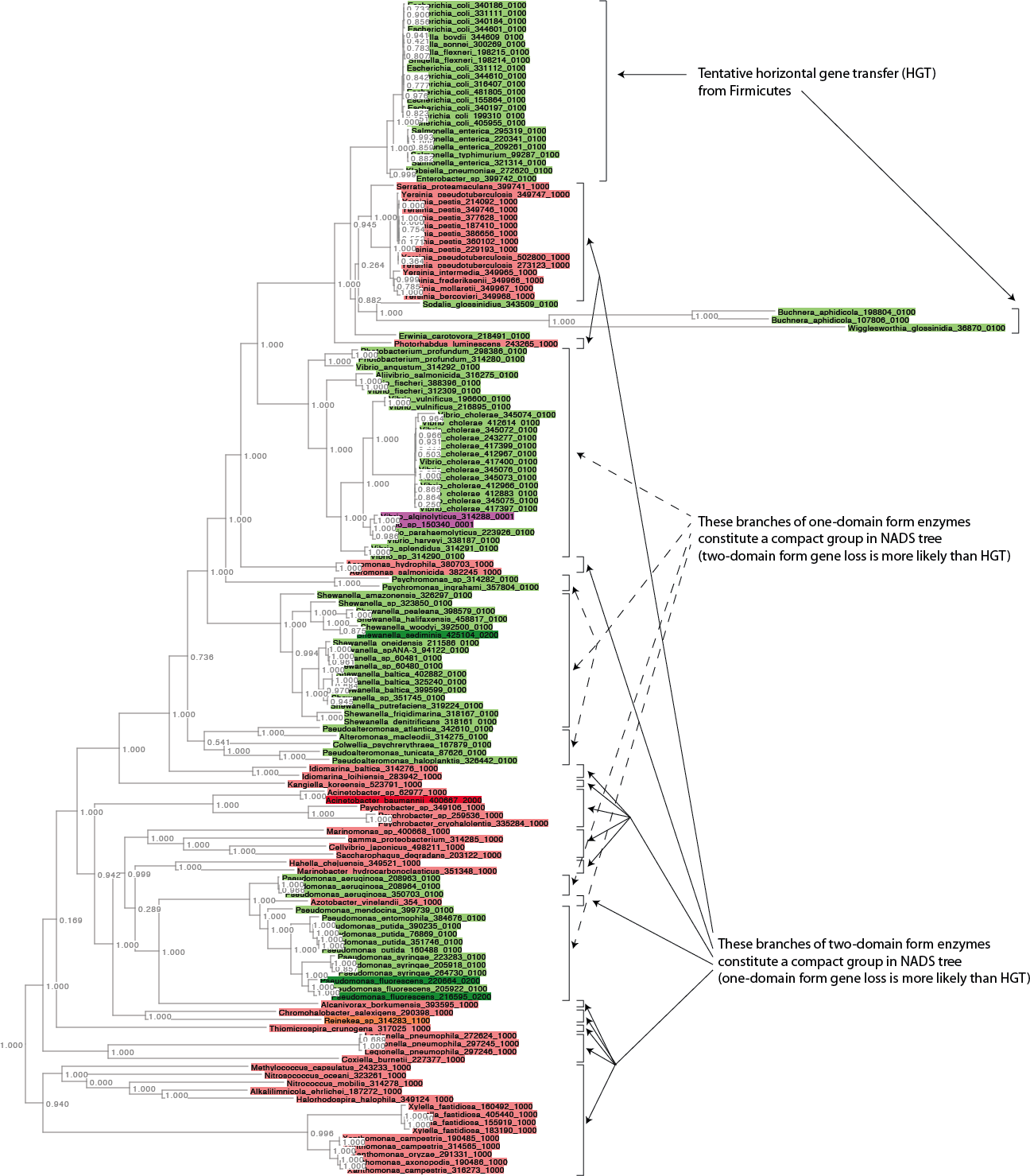

Supplement: Figure S7 — The γ-proteobacteria branch of species tree annotated with suggested HGT and gene loss events. (TIF) [file pone.0039115.s007.tif]
