# Supplementary material for: Glutamine versus Ammonia Utilization in the NAD Synthetase Family
Source: PLoS One. 2012 Jun 15;7(6):e39115. doi: 10.1371/journal.pone.0039115 (PMC3376133; doi:10.1371/journal.pone.0039115)
Supplement: Table S1 — Bacterial strains and plasmids used in this study. (DOCX) [file pone.0039115.s008.docx]

**Table S1. Bacterial strains and plasmids used in this study**

| **Strain or plasmid** | **Description** | **Reference or Source** |
| --- | --- | --- |
| *E. coli* |  |  |
| BL21(DE3) | Used for protein expression | Novagen |
| DH5α | Used for recombinant DNA methods | Invitrogen |
| *S. typhimurium* |  |  |
| LT2 | Parent Salmonella strain | [34], This Study |
| TA1650 | Parent Salmonella strain | [34], This Study |
| Nit11 | Salmonella *nadE* mutant strain carrying a missense mutation (S48N) | [34], This Study |
| SK51 | Salmonella *nadE* mutant strain carrying single nucleotide (guanine) deletion in the promoter region ( pos. -51)^(a)^ | [34], This Study |
| pET15b | IPTG-inducible expression vector; Ap^r^ | Novagen |
| pODC | pET-derived vector IPTG-inducible expression vector; Ap^r^ | [35] |
| pET15b_nadE | *Salmonella typhimurium* *nadE* inserted between NdeI and BamHI sites of pET15b | This study |
| pET15b_nadE^nit11^ | *nadE* from *S. typhimurium* nit11 mutant inserted between NdeI and BamHI sites of pET15b | This study |
| pODC_ TTC1538-9 | *Thermus thermophilus* operon region encompassing  NAD synthetase and glutaminase genes inserted between NcoI and SalI sites of pET-derived vector pODC | This study |
| pODC_TTC1538 | *Thermus thermophilus* S-subunit (nadE) inserted between NcoI and SalI sites of pET-derived vector pODC | This study |
| pODC_TTC1539 | *Thermus thermophilus* G-subunit inserted between NcoI and SalI sites of pET-derived vector pODC | This study |

^(a)^ Considering -1 the first base upstream of the first codon
