# Supplementary material for: Glutamine versus Ammonia Utilization in the NAD Synthetase Family
Source: PLoS One. 2012 Jun 15;7(6):e39115. doi: 10.1371/journal.pone.0039115 (PMC3376133; doi:10.1371/journal.pone.0039115)
Supplement: Table S2 — Primers used for gene sequencing, cloning, and qRT-PCR. (DOCX) [file pone.0039115.s009.docx]

**Table S2. Primers used for gene sequencing, cloning, and qRT-PCR**

| **Use** | **Direction** | **Sequence 5'🡪3'** |
| --- | --- | --- |
| Sequencing of *st*_NADS gene | Forward | agcgtgttatgtaagcctgga |
|  | Reverse | aaaaaagaggctgggaagag |
| Amplification of *st*_NADS for rtPCR | Forward | aatcgctggtgttaggcatc |
|  | Reverse | cgataaactgcagcgcatta |
| Amplification of *st*_gapA for rtPCR | Forward | gacaaatacgaaggccagga |
|  | Reverse | cggtagtcgcgtgaacagta |
| Cloning of *st*_NADS into pET15b | Forward | ttttttcatatgactctgcagcaagaga |
|  | Reverse | gtagaaggatccttaccttttccaaaagtc |
| Cloning of *st*_NADS^nit11^ into pET15b | Forward | ttttttcatatgactctgcagcaagaga |
|  | Reverse | gtagaaggatccttaccttttccaaaagtc |
| Cloning of *tt*_NADS_S/G into pODC | Forward | gggtcatgaccgaggtgcgccacgccgtcc |
|  | Reverse | ggggtcgacctaagggcggtagtccaagggcctcaggtagaac |
| Cloning of *tt*_NADS_S into pODC | Forward | gggtcatgaggctgcagatacttgaagcgcccaaagcccaggaggtcc |
|  | Reverse | ggggtcgacctaagggcggtagtccaagggcctcaggtagaac |
| Cloning of *tt*_NADS_G into pODC | Forward | gggtcatgaccgaggtgcgccacgccgtcc |
|  | Reverse | ggggtcgactcactgcagagtatccgcatcccgcctccctaagaccctttg |
| Cloning of *mj*_NADS into pODC | Forward | atttccacatgttatggggtgagagtatg |
|  | Reverse | ctaaaatgtcgacttaaatctctggtgttgg |
