# Supplementary material for: Glutamine versus Ammonia Utilization in the NAD Synthetase Family
Source: PLoS One. 2012 Jun 15;7(6):e39115. doi: 10.1371/journal.pone.0039115 (PMC3376133; doi:10.1371/journal.pone.0039115)
Supplement: Text S1 — Correlation between predicted glutamine-utilizing property and glutamine-utilizing signature motifs. (DOCX) [file pone.0039115.s012.docx]

**Correlation between predicted glutamine-utilizing property and Gln-utilizing signature motifs**.

In the terms of branches that were defined using NADS phylogenetic tree, we found that identified set of structural elements – α9, α18 helices and extended C-terminal loop - are uniformly present in eukaryotic and bacterial two-domain form of NAD synthetase (branches I-III in **Figure 5A**) and absent in bacterial one-domain form of enzyme (branches IV-VII). A striking correlation was observed for the bacterial and archael branches IV and V, respectively, both containing a mix of the glutamine- and ammonia-utilizing evolutionary close enzymes. Thus, the glutamine-utilizing enzymes from these groups, while being similar to ammonia-utilizing enzymes from the same groups by sequence, universally contains the identified structural elements in contrast to ammonia-utilizing enzymes from these groups, among which there is no case of presence of the intact set of identified structural elements. Interestingly, the largest component of these structural elements - extended C-terminal loop – possess a similar sequence of amino acids in evolutionary distinct bacterial and archaeal branches IV and V. Because enzymes having extended C-terminal loop from these groups were evolved independently from one-domain form, as we concluded in evolutionary scenario, they represent a remarkable case of convergent evolution. We suggest that the formation of complex with glutaminase domain can be explained as essential part of the emergence of glutamine-utilizing ability of NAD synthetase due to the necessity for formation of intersubunit ammonia tunnel, as observed in NAD synthetase structures from *Mycobacterium tuberculosis* [1,2].

1. Chuenchor W, Doukov TI, Resto M, Chang A, Gerratana B (2012) Regulation of the intersubunit ammonia tunnel in Mycobacterium tuberculosis glutamine-dependent NAD+ synthetase. Biochem J 443: 417-426.

2. LaRonde-LeBlanc N, Resto M, Gerratana B (2009) Regulation of active site coupling in glutamine-dependent NAD(+) synthetase. Nat Struct Mol Biol 16: 421-429.
