# Supplementary material for: Glutamine versus Ammonia Utilization in the NAD Synthetase Family
Source: PLoS One. 2012 Jun 15;7(6):e39115. doi: 10.1371/journal.pone.0039115 (PMC3376133; doi:10.1371/journal.pone.0039115)
Supplement: Text S2 — The mosaic distribution of one and two-domain NAD synthetase in Eubacteria. (DOCX) [file pone.0039115.s013.docx]

**The mosaic distribution of one and two-domain NAD synthetase in Eubacteria.**

There are several types of evolutionary events that theoretically could have led to observed mosaic distribution of two forms of enzyme in Eubacteria (**Figure 5, Figure S1, and Figure S4**). These events include the domains fusion/fission, horizontal gene transfer and differential gene gain/loss. We analyzed the impact of each type of event on the γ-proteobacteria and Deinococcus-Thermus groups, focusing on the two representative members, *Salmonella typhimurium* and *Thermus thermophilus* that were experimentally studied in this work. On the basis of the NADS tree analysis, earlier in this section we have excluded the possibility of domains fusion/fission events on the course of evolution of eubacteria, except for one initial fusion event that took place in the early stages of evolution, presumably before the origin of LECA, and gave birth to the two-domain form of NAD synthetase enzyme. Then, we reconstructed possible HGT and gene gain/loss events by applying the method of searching of topology inconsistencies in the species and gene trees (1). The γ-proteobacteria branch of species tree annotated with suggested HGT and gene loss events is depicted in **Figure S7**. The whole branch consists of alternating patches (subbranches) of bacteria having one and two domain NAD synthetase forms. Bacteria from the 14 two-domain form subbranches represent a compact cluster in the NADS tree. This points out for an existence of two-domain form gene in the ancestor of γ-proteobacteria. On the other hand, all 7 one-domain form subbranches, except one to which *S. typhimurium* belongs, also comprise a compact group in the NADS tree. Thus, we speculate that ancestor of γ-proteobacteria possibly had both one- and two-domain forms and the observed mosaic distribution is mostly the results of respective loss of either form of the enzyme. The single subgroup of organisms from *Salmonella*'s one-domain form sub-branch that resides distantly from another γ-proteobacteria on NADS tree is presumably originated from horizontal gene transfer. Our analysis of homology of Salmonella’s group to other NAD synthetase enzymes suggests that the ancestor of Salmonella's sub-branch came from Firmicutes. The Deinococcus-Thermus group contains a small number of bacteria having complete genomes and all of them possess one-domain form of NAD synthetase enzyme. However, these enzymes are distributed in the NADS gene tree into two distant clusters. The cluster of Deinococcales NAD synthetase seems to be originated from the Deinococcus-Thermus group ancestral enzyme. We suggest it from the analysis of the adjacent to Deinococcus-Thermus group branches in the species tree. Thus, the most populated among other neighbors Actinobacteria group contains one-domain form enzymes that resides in this cluster only. The second cluster consisting of enzymes from Thermales order includes *T. thermophilus* NAD synthetase enzyme. A larger number of enzymes from this cluster are located in other taxonomic groups: Deltaproteobacteria, Deferribacteres, Firmicutes, Fusobacteria, Chlorobi, Chloroflexi. This fact suggests that existence of these enzymes in Deinococcus-Thermus is presumably the result of horizontal transfer, although the exact source of the transfer is difficult to resolve. On the basis of presented analysis of the γ-proteobacteria and Deinococcus-Thermus groups we conclude that both horizontal transfer and gene loss, but not the fusion or fission events played a central role in the evolution of NAD synthetase enzyme in eubacteria.

1. **Gogarten, J. P., and J. P. Townsend.** 2005. Horizontal gene transfer, genome innovation and evolution. Nature reviews Microbiology **3:**679-87.
